# Research Progress of DNA Methylation in Endometrial Cancer

**DOI:** 10.3390/biom12070938

**Published:** 2022-07-04

**Authors:** Ting Xu, Hongmei Ding, Jie Chen, Jiahui Lei, Meng Zhao, Bingyu Ji, Youguo Chen, Songbing Qin, Qinqin Gao

**Affiliations:** 1 Institute for Fetology, The First Affiliated Hospital of Soochow University, Suzhou 215006, China; cynthia_tingxu@126.com (T.X.); snailnail@163.com (J.L.); z772082338@126.com (M.Z.); susanji0000@126.com (B.J.); 2 Department of Obstetrics and Gynecology, The First Affiliated Hospital of Soochow University, Suzhou 215006, China; ccb1986@126.com (H.D.); chen_41750841@126.com (J.C.); 3 Department of Radiation Oncology, The First Affiliated Hospital of Soochow University, Suzhou 215006, China

**Keywords:** DNA methylation, tumor suppressor genes, oncogenes, endometrial cancer

## Abstract

Endometrial cancer (EC)) is one of the most common malignant tumors of the female genital system, with an increasing incidence and mortality, worldwide. Although the therapeutic strategy of EC is still complicated and challenging, further understanding of carcinogenesis from a gene perspective would allow an effort to improve therapeutic precision in this complex malignancy. DNA methylation is the most widely studied epigenetic alteration in human tumors. Aberrant DNA methylation events, resulting in altered gene expression, are features of many tumor types. In this review, we provide an update on evidence about the roles of aberrant DNA methylation within some classical tumor suppressor genes and oncogenes in endometrial carcinogenesis, and report on recent advances in the understanding of the contribution of aberrant DNA methylation to EC, as well as opportunities and challenges of DNA methylation in EC management and prevention.

## 1. Introduction

Endometrial cancer (EC) is one of the most common malignant tumors of the female genital system, and its incidence is rising globally [1,2]. The incidence and trend of EC have been suggested to vary by ethnicity and geographical region. It is generally recognized that the incidence of EC is higher in developed countries than in developing countries [1,3]. EC is most commonly found in perimenopausal and postmenopausal women. After menopause, the ovaries stop synthesizing estrogen and progesterone. There is more unopposed extra-glandular production of estrogens, which results in unbalanced proliferation of endometrium and has been associated with an increased risk of EC for a long time [3]. In addition to estrogen, there is a series of risk factors for EC, such as obesity, increasing age, family history or genetic predisposition, early menarche, late menopause, polycystic ovarian syndrome, and diabetes [3,4]. ECs are usually classified into two subtypes: type I and type II. Type I ECs, also known as endometrioid ECs (EECs), are low grade, estrogen dependent with endometrioid morphology that account for roughly 85% of all instances of ECs. They are frequently detected early and have a favorable prognosis. Type II ECs are characterized by non-endometrioid subtypes, such as serous and clear cell carcinomas, usually high grade, hormone receptor negative, and associated with a bad prognosis [4,5]. Surgery (total hysterectomy, bilateral salpingo-oophorectomy, and lymphadenectomy) is the primary treatment for most EC patients, whether type I ECs or type II ECs [4]. The optimal courses of treatment for EC patients with uterine-confined disease that are not amenable to initial surgery are radiation therapy and/or brachytherapy [6]. Chemotherapy is frequently chosen for patients with grade 1 or 2 hormone receptor-positive tumors that are not aggressively progressing [4]. For individuals with grade 1, stage IA noninvasive EC who desire fertility sparing, hormonal treatment is the preferred choice [6,7]. EC patients who have recurring low-grade endometrioid histology may benefit from hormonal therapy [8]. There are some differences between the treatment strategies for type I and type II ECs. For instance, in patients with type I ECs, adjuvant radiotherapy is not suggested in some conditions. However, for patients with type II ECs, adjuvant radiotherapy is recommended in the majority of cases, possibly because most type II ECs are high-risk ECs [4,9].

ECs are not readily explained or caused by a single element. The molecular abnormalities in the two types of ECs are different. Microsatellite instability (MSI) and abnormalities in the PTEN, K-RAS, PIK3CA, and β-catenin genes are common in Type I ECs, but Type II ECs frequently show extensive nuclear atypia and aberrant p53 staining [3,10]. The intracellular pathways that regulate gene expression are diverse and complex, such as gene point mutation, variation in gene copy number, and epigenetic regulation. In recent years, there has been a growing consensus that epigenetic mechanisms play vital roles in tumor development and progression [11,12]. DNA methylation alteration is one of the most important epigenomic changes, resulting in aberrant expression of tumor-related genes in various human tumors [11,12]. DNA methylation is catalyzed by DNA methyltransferases and occurs in the mammalian genome. Cytosine methylation of DNA within the CpG dinucleotide is the most well-researched epigenetic alteration in human beings. It is reported that in normal human DNA, 4–6% of all cytosines are methylated [13]. CpG dinucleotides are frequently clustered in CpG islands. These islands are usually in or around the promoter regions and are often unmethylated in normal tissue [14]. There is accumulating evidence that abnormal patterns of DNA methylation have been reported to be recognizable in various cancers [11,15]. Similar to other types of cancer, EC cells acquire two main types of aberrant DNA methylation patterns during malignant transformation: local DNA hypermethylation and global DNA hypomethylation [16]. DNA hypermethylation is associated with the promoter regions of mainly tumor suppressor genes, whereas DNA hypomethylation can occur in normally methylated DNA sequences and leads to upregulation of many genes, especially oncogenes [17]. Currently, numerous methylation studies have reported that more than 50 hypermethylated tumor suppressor genes and several hypomethylated oncogenes have been identified in ECs [16,18]. These genes with aberrant DNA methylation are involved in perturbing various biological pathways, such as cell adhesion, cell proliferation, cell cycle regulation, and also apoptosis, which ultimately contribute to the development and progression of ECs [16,18]. In the following section, we specifically discuss DNA methylation alterations in these genes in endometrial carcinogenesis and summarize recent advances in the understanding of the contribution of aberrant DNA methylation to ECs, as well as opportunities and challenges of DNA methylation in EC management and prevention (Table 1).

## 2. Tumor Suppressor Genes

### 2.1. RASSF1A (Ras Association Domain Family 1)

The RASSF1 gene is located on chromosome 3p21.3 and expresses eight main transcript variants through alternative splicing [56,57]. The major, ubiquitous transcripts are *RASSF1A* and *RASSF1C*. *RASSF1A* and *RASSF1C* have similar structural characteristics; however, these two isoforms play opposite roles during carcinogenesis [57]. *RASSF1A* is a tumor suppressor that promotes apoptosis while inhibiting proliferation, migration, and invasion, whereas *RASSF1C* is an oncogene with the opposite functions [57]. RASSF1A functions as a scaffolding protein, allowing numerous effector protein complexes to be assembled and modulated, and is located at the core of the signal transduction network that includes cell cycle and apoptotic signaling modules [58].

In 2000, Dammann et al., first reported that the loss of expression of RASSF1A in lung cancer was associated with methylation of its CpG-island promoter [59]. Numerous research studies have demonstrated the abnormal expression of RASSF1A in different kinds of tumors, such as nasopharyngeal carcinoma, breast cancers, and ECs [60,61,62]. The overwhelming majority of reports revealed that the CpG island in the *RASSF1A* promoter was hypermethylated, which has been linked to the malignant transition of early benign tumors into later invasive carcinomas [63,64]. According to Pallare et al., ECs had significantly lower RASSF1A expression than normal endometrium; EC samples with RASSF1A-negative immunostaining showed higher *RASSF1A* promoter methylation (96%) [19]. Several studies reported that treating human EC cell lines with 5-aza-2’-deoxycytidine could restore RASSF1A expression and reverse *RASSF1A* promoter methylation [65,66]. *RASSF1A* methylation was observed at a higher frequency in ECs than in others; the frequency of *RASSF1A* methylation ranged from 33% to 85% [20,21,65,67] (Table 2 and Table 3). Kang et al., observed that RASSF1A gene methylation was found in 81% of endometrial adenocarcinomas, while just 33.3% was detected in cervical adenocarcinomas [22]. Arafa et al., showed that in 74% (29/39) of EEC samples and 36% (4/11) of normal endometrial tissues adjacent to EEC, the RASSF1A gene was methylated [23]. Similar to Arafa, data from Pallare et al., suggested that the frequency of *RASSF1A* promoter hypermethylation was 74% (42/57) in EC samples [19]. Besides, Zhang et al., reported that the *RASSF1A* promoter methylation was more frequent in EC tissue than in adjacent normal endometrial tissue, which was 57% (20/35) and 40% (6/15), respectively [24]. Multinu et al., reported a consistent conclusion that the percentage of *RASSF1A* methylation increased as benign endometrial progressed to EC [68].

The above studies provided evidence that *RASSF1A* promoter methylation could be an early event in ECs. A meta-analysis conducted by Pabalan et al., showed that the *RASSF1A* promoter methylation was strongly associated with increased EC risk. In their research, *RASSF1A* promoter methylation was 11-times more likely to predict ECs in women [77]. Liao et al., reported that 56.6% (43/76) of EC showed aberrant hypermethylation of *RASSF1A*, and there was a significant difference between type I and type II ECs, which was 61.5% and 27.2%, respectively [66]. Besides, Jo et al., showed that patients with *RASSF1A* hypermethylation had a substantially poorer 5-year disease-free survival than those who did not have methylation [25].

As we all know, tissue biopsies are still used often in EC diagnosis and screening. They are intrusive, time consuming, and not consistently ideal for screening. On the other hand, liquid biopsy, such as blood, urine, and secretion of patients, is a noninvasive diagnostic process that allows for early tumor diagnosis. Fiegl et al., first reported that DNA methylation of *RASSF1A* was observed in the cervicovaginal secretion of patients with ECs. The DNA methylation of *RASSF1A* in cervicovaginal secretion was assessed by MethyLight (a fluorescence-based, real-time PCR assay). In their study, three or more gene methylations of the five genes (including *RASSF1A*) were established in all EC patients, and the specificity of this method for detecting EC patients was 97.2% [78]. Kim et al., found that the methylation of *RASSF1A* in cervical scrapings exhibited a specificity of 96.3% for EC detection [79]. These studies indicate that quantitative detection of aberrant DNA methylation in cervical scrapings may be a promising new diagnostic tool for the detection of ECs. Recently, aberrant methylation of the *RASSF1A* promotor was also found in epithelial ovarian carcinoma patients’ plasma samples [80]. It is not surprising to us that *RASSF1A* promoter methylation is an intriguing biomarker for EC detection because of its distinctive characteristics.

### 2.2. p16

*p16* belongs to the INK4 (inhibitor of cyclin-dependent kinase 4) family, which includes *p16^INK4A^*, *p15^INK4B^*, and *p19^INK4D^* [81]. *p16* has several names, such as *p16 ^INK4A^*, *MTS-1* (major tumor suppressor 1), and *CDKN2A* (cyclin-dependent kinase inhibitor 2A). The *p16* gene maps to human chromosome 9p21.3. Since *p16* was first discovered in 1993, great progress has been made in establishing *p16* as a tumor suppressor and delineating the *p16*/CDK/cyclin D pathway as a key regulator of cell proliferation [81,82]. It was well established that the main biochemical function of *p16* is to stop cell cycle progression at the G1 to S phase, and *p16* deficiency could definitely contribute to cancer development by enabling uncontrolled cellular proliferation [83].

A considerable amount of evidence showed that, in addition to mutations and homozygous deletions, frequent promoter methylation of the *p16* gene, which results in transcriptional silencing, is a key event in the development of EC [26,84]. Guida et al., utilized the MSP assay (methylation-specific PCR) to determine the methylation status of the *p16* gene and found that aberrant methylation of *p16* was seen in 75% of EC, and there was an increased trend of *p16* hypermethylation along with the development of diseases [27]. Cornel et al., reported that *p16* promoter methylation was in 38% of EC patients, which was significantly higher than in atypical hyperplasia endometrial (7.7%) [28]. Ignatov et al., reported that promoter hypermethylation of *p16* in EC patients was 17.4% [29]. It is interesting to note that Yang et al., reported that 25% of *p16* methylation was present in EC. What was unexpected is that none of the 14 stage II–IV carcinomas were found to have *p16* gene hypermethylation [70]. Contrary to the above reports, in Seeber et al., and other independent research, the frequency of *p16* promoter methylation was less than 5% in EC; the method for measuring DNA methylation used in the study of Seeber et al., was MS-MLPA (methylation-specific multiplex ligation-dependent probe amplification), which was different from other studies [20,35,85]. The study by Zhang et al., showed that there was no significant difference in *p16* promoter methylation between the EC tissue and adjacent normal endometrial tissue, which was 37% and 27%, respectively [24]. Additionally, Yanokura et al., reported that a significant frequency of hypermethylation in the promoter region of the *p16* gene was seen in five out of eight EC cell lines, but hypermethylation was not seen in any of the 32 endometrial carcinoma specimens from Japanese patients [69].

In short, the reported methylation rates of *p16* were not completely consistent in EC. The potential causes of the discrepancy between different studies could be the methods used for research were different or the heterogeneity of the population investigated in the different studies. Numerous meta-analysis studies demonstrated that *p16* gene hypermethylation was significantly correlated with EC patients [86,87]. By evaluating the methylation status of the *p16* gene between patients, Chao and co-workers found that hypermethylation of the *p16* gene was highly correlated with inhibition of *p16* gene transcription, which was associated with the progression of endometrial carcinoma [71]. Besides, Chao et al., observed that after exposure to 5-aza-2’-deoxycytidine, the methylation level of the *p16* gene was gradually reduced, and *p16* mRNA expression was restored in endometrial carcinoma xenografted in nude mice [88]. Although more research about the association between methylation of *p16* and ECs needs to be further investigated and confirmed, the therapeutic potential of restoring tumor suppressor *p16* expression by DNA methylation might provide fresh hope for EC treatment through gene-targeted therapy.

### 2.3. hMLH1 (Human MutL Homolog 1)

Lynch syndrome is an autosomal dominant genetic disorder that accounts for a certain proportion of all EC [89]. Thus, it is essential to explore the mechanism of Lynch-syndrome-associated EC. This syndrome is caused by a germline mutation of the DNA mismatch repair (MMR) genes [90]. Several studies have consistently found that in patients with Lynch-syndrome-associated colorectal cancer, germline mutations in *hMLH1* (one of the MMR genes) account for a considerable number of cases [91,92]. Evidence showed that patients with *hMLH1* variants had a high cumulative incidence of any Lynch-syndrome-associated cancer [93]. In addition, a faulty MMR system could result in MSI and, eventually, carcinogenesis by altering genes associated with cell regulation proteins [90].

MSI has also been found in around 20% of sporadic EC and around 40% of EEC [94,95]. In a prospective and population-based endometrial carcinoma study, 93% of MSI-positive tumors showed loss of nuclear expression of *hMLH1* [96]. On the other hand, Esteller et al., discovered that *hMLH1* promoter hypermethylation and MSI were present in 11 of 12 (91%) instances of endometrial carcinoma [30]. An independent study by Pauly et al., demonstrated that there were 81.4% (22 out of 27) of endometrial carcinoma patients who had hMLH1 loss or microsatellite instability and showed *hMLH1* promoter hypermethylation [31]. The aberrant expression of hMLH1 is undoubtedly linked to MSI, and DNA methylation of *hMLH1* plays a critical role in the development of MSI. Several studies showed that the *hMLH1* methylation frequency in EC had a significant association with the loss of its protein level [96,97]. Additionally, Xiong et al., indicated that in EC patients with relatively low hMLH1 mRNA expression, 7 to 11 samples were found to contain a completely methylated *hMLH1* promoter [32]. Additionally, Domenico et al., showed that *hMLH1* hypermethylation was 84.6% in EEC, and there was a similar percentage in the peritumoral endometrium [26]. The data above demonstrated that *hMLH1* hypermethylation is a common and early event in EC tumorigenesis.

Otherwise, research by Whitcomb et al., showed the frequency of *hMLH1* promoter methylation was 33.3% in primary endometrial tumors, but there was no significant difference between primary endometrial tumors with or without recurrence [72]. Furthermore, Bischoff et al., showed that *hMLH1* promoter methylation was detected in 24 out of 64 (37.5%) primary EC and in 1 out of 18 (5.6%) metastatic tissues [98], confirming the opinion that *hMLH1* promoter hypermethylation occurs in early endometrial carcinoma. Borden et al., recently reported that *hMLH1* hypermethylation in EC had a strong association with higher recurrence rates and worse prognosis; *hMLH1* promoter hypermethylation in individuals was more likely to recur, even in patients accompanied by low-grade, early-stage cancer [99]. In addition, Seeber et al., discovered 26% *hMLH1* promoter methylation in EEC, and *hMLH1* methylation was strongly associated with shorter disease-free survival and overall survival in EEC [20]. Besides, Yang et al., demonstrated only 13.3% *hMLH1* promoter methylation was detected in EC, and this alternation was more prevalent in poorly differentiated tumors than in well-differentiated cancers [70]. It is obvious that patients with *hMLH1* hypermethylation usually have poor prognostic features. The connection between *hMLH1* hypermethylation and outcomes in EC needs to be further explored. Yang et al., found that after 72 h of treatment with RG108 (a demethylation drug), the amount of methylated hMLH1 gene was significantly reduced in EC cell lines [100]. Similar results have been seen in 5-aza-2’-deoxycytidine [101]. In conclusion, hypermethylation of the *hMLH1* promoter is useful in early disease diagnosis, even though its potential value in disease progression warrants further investigation. These findings may aid in the development of novel and effective treatments for endometrial cancer by restoring hMLH1 expression and related DNA repair activity. Recently, Loukovaara et al., reported that the response to adjuvant therapy of EC patients could be predicted by the MMR protein and *hMLH1* methylation status. In MMR-deficient nonmethylated tumors, but not in *hMLH1* methylated tumors, both whole-pelvic radiotherapy and chemotherapy combined with radiotherapy were associated with poor disease-specific survival [102]. More studies are needed to confirm and reveal the effects of gene methylation on the response of patients to treatments.

### 2.4. PTEN (Phosphatase and Tensin Homolog)

The PTEN gene is located on chromosome 10q23 and encodes a 403-amino acid that acts as a lipid and protein phosphatase. *PTEN* is the most generally used name, also known as *MMAC1* or *TEP1*. *PTEN* has been identified in numerous tumors [103,104]. In 1997, two independent research groups, Risinger et al., and Tashiro et al., first reported that *PTEN* was a tumor suppressor gene in EC [103,105]. The PI3K/AKT/mTOR signaling axis is mainly involved in tumor cell growth, proliferation, and motility and has been shown to participate in the production and progression of EC [106]. *PTEN* has been shown to function as a tumor suppressor phosphatase, negatively regulating this signaling pathway [107].

Various studies indicate that PTEN is one of the most frequently changed genes in EC. Although *PTEN* was found to be more frequently mutated in EC, alterations in PTEN expression have been suggested to play a contributing role in the pathophysiology of EC progression [108]. Mutter and colleagues provided evidence that loss of PTEN function was an early event in endometrial tumorigenesis [109]. Additionally, Zhang et al., reported that the expression of PTEN was significantly decreased in EC and had a tight correlation with the progression of this disease. Using the expression of PTEN mRNA to diagnose EC, the sensitivity and specificity were 85.3% and 83.6%, respectively [110]. According to Salvesen et al., during endometrial carcinogenesis, gene mutation and methylation may both work to inactivate PTEN expression and their data also showed that the presence of the *PTEN* mutation was always linked to a favorable prognosis. [111]. Seeber et al., mentioned the low frequency of *PTEN* methylation in EEC [20]. However, an increasing number of studies indicated that the DNA methylation within the promoter of the PTEN gene was commonly observed and relatively frequent in EC [33,34]. Salvesen et al., demonstrated that the methylated *PTEN* promoter region was found in 26 of 138 (19%) EC patients, and *PTEN* methylation was associated with the advanced, metastatic, and MSI phenotypes in EC [33]. In addition, Ghazanfari et al., observed that promoter hypermethylation of *PTEN* was in 28.57% of EC patients. They also found that *PTEN* methylation levels in patients’ blood increased significantly (11.54%) compared to normal tissue (4.54%) [34]. The present study tends to indicate that the promoter hypermethylation of *PTEN* is a risk factor in EC.

Although there are limited studies on *PTEN* methylation alterations in EC, the importance of *PTEN* promoter methylation on endometrial tumorigenesis should not be ignored. Recently, regulating *PTEN* promotor methylation has been reported to play an important role in the progression of EC. The research of Yi et al., demonstrated that Linc00470 recruited DNMT3a through MYC to promote *PTEN* methylation and promote angiogenesis and metastasis of EC cells in vivo [112]. Chen et al., demonstrated that Piwil1 repressed PTEN expression through DNMT1-mediated *PTEN* hypermethylation in type I ECs [113]. Collectively, these studies provide a new view of potential therapeutic targets for treatment of EC.

### 2.5. APC (Adenomatous Polyposis Coli)

*APC* has a role in carcinogenesis in a variety of cancers, such as gastrointestinal cancers, breast cancer, and EC [36,114]. Wnt/β-catenin signaling is generally associated with organ development, cell proliferation, survival, differentiation, and migration [115,116]. APC is a key component of the Wnt/β-catenin signaling transduction pathway’s destruction complex [116]. According to a growing body of evidence, Wnt/β-catenin signaling disruption has been linked to the formation and/or progression of malignancies, including ECs [116,117].

Research indicates that *APC* promoter methylation rates in EC vary. Some studies reported *APC* promoter methylation occurred at around 20% of the frequency [34,37], while others showed the frequency was nearly 40% [35,73]. DNA methylation of the APC gene is unquestionably linked to the prevalence and progression of EC. Zysman et al., first reported that *APC* methylation was detected in EC and demonstrated that *APC* methylation was shown to be more strongly related to MSI status [38]. Beyond that, Moreno-Bueno et al., revealed the methylation status of the *APC* promoter in 103 ECs and found promoter hypermethylation in 48 of 103 (46.6%) [39]. Furthermore, Qian et al., found that in endometrioid adenocarcinoma, the methylation rate of the APC gene was much greater than in atypical hyperplastic endometrium and normal proliferative endometrium (65.0%, 33.3%, and 23.3%, respectively). *APC* methylation had a significant association with the low expression of APC mRNA and proteins detected in EC [40]. Moreover, research by Banno et al., found that the frequencies of aberrant methylation of the APC gene in atypical hyperplasia and EC were 7.2% and 22%, respectively [37]. In addition, Ignatov et al., reported that hypermethylation of the APC gene promoter was discovered in atypical hyperplasia and in early EC (23.5% and 56.9%, respectively); the incidence of hypermethylation in the *APC* promoter decreases as EC progresses [36]. These data confirmed the opinion that *APC* promoter methylation is an early and crucial event in the progression of EC. However, other studies discovered that *APC* promoter methylation was more common in patients with atypical hyperplasia (42.4%) than in patients with EC (19.6%) [28]; methylation status was not significantly different between different clinicopathological characteristics [70]. This disparity might be attributable to a different sample size, different methods used for comparison, and the heterogeneity of the population investigated in different studies. Recently, some microRNAs were discovered that influence APC expression by changing the state of the APC gene promoter methylation [118], providing the important idea of DNA methylation-related drugs for the treatment of EC.

### 2.6. E-Cadherin (Epithelial Cadherin, Also Known as CDH1)

The E-cadherin gene is located on chromosome 16q22.1 and belongs to the calcium-dependent cell–cell adhesion molecule and tumor suppressor protein [119,120]. Because of its early discovery and detailed characterization in both normal and pathological tissues, it is often regarded as the archetype of all cadherins. E-cadherin mediates contact inhibition of proliferation and, thus, plays an important role in cell growth and proliferation [119,120]. Otherwise, E-cadherin on the cell surface could bind to β-catenin and trap it at the membrane, inhibiting Wnt signaling and blocking β-catenin nuclear translocation [120]. In normal endometrial tissue and most of the atypical hyperplastic endometrial tissue, E-cadherin showed primarily epithelial membranous reactivity with a homogeneous pattern of distribution. In EC cells, redistribution of E-cadherin reactivity was observed to be primarily membranocytoplasmic, with a heterogeneous pattern of distribution [121]. Alterations in E-cadherin membrane expression promote carcinogenesis and lead to increased invasiveness and a metastatic process in EC [122].

E-cadherin gene methylation is a common event in EC patients [22,36,41]. Five of ten human endometrial adenocarcinoma cell lines showed methylation alternation in the E-cadherin gene, and the mRNA expression of E-cadherin in the corresponding cell line was reduced or even negative [123]. Yi et al., found E-cadherin gene promoter methylation was significantly higher in the EC group but not detected in the normal endometria or atypical hyperplasia endometria groups [76]. They further claimed that methylation of the E-cadherin gene promoter reduced the expression of E-cadherin and lowered the overall 5-year survival rate [76]. Similar to Yi et al., Banno et al., reported that the incidence of hypermethylation of *E-cadherin* was 14% in EC, and this methylation alternation was not detected in normal endometrium or atypical endometrial hyperplasia [37]. Additionally, E-cadherin gene promotor hypermethylation showed a tendency for higher tumor grade, deeper myometrial invasion, and local lymphatic metastasis [42]. However, Fiolka et al., reported that promoter methylation of the E-cadherin gene was 31.4% in EEC samples, 21.1% in endometrial complex hyperplasia cases, and 20.0% in healthy endometrium; there was no distinction among different histologic categories [67]. Instead, several studies showed that E-cadherin gene promoter methylation was not detected in EC [74,75]. The relationship between methylation in the E-cadherin gene promoter region and EC is not consistent. Further research on the involvement of *E-cadherin* promoter methylation in endometrial carcinogenesis is necessary. What is clear is that the treatment of endometrial epithelial carcinoma cells with 5-aza-2’-deoxycytidine could increase the expression of E-cadherin [124,125]. These data presented above provide a new potential method for conducting mechanistic studies of *E-cadherin* in EC as well as a new therapeutic strategy for EC.

### 2.7. Other Tumor Suppressor Genes

Other tumor suppressor genes whose methylation changes contribute to endometrial carcinogenesis include *CDH13* (Cadherin-13), *ESR1* (Estrogen receptor 1, encoded estrogen receptors), *O6-MGMT* (O6-methylguanine-DNA methyltransferase), *PRs* (Progesterone receptors, including *PRA* and *PRB*), *RAR2* (Retinoic acid receptor 2), and so on. The CDH13 gene methylation frequency in most studies was high, with 60% to 90% [20,35,43]. Sheng et al., claimed CDH13 gene methylation alternation in EC played an important role in the early stage; the percentage of CDH13 gene methylation in normal endometrium, endometrial hyperplasia, and EC was 14.81%, 40.5%, and 81.36%, respectively [44] (Table 3). Sasaki et al., first reported that the CpG methylation pathway inactivates ESR1 gene promoter C in EC [45]. Recently, studies by Dan et al., demonstrated that ESR1 and PR genes were hypermethylated in 41.1% and 24.8% of EEC, respectively [46]. The research of Rimel et al., reported that in EC, methylation of the O6-MGMT gene promoter was a rare occurrence [126]. In contrast to this, Suehiro et al., reported that the frequency of hypermethylation of the O6-MGMT gene in EC was 8.6% [35]. Additionally, Cornel and coworkers found the frequency of O6-MGMT gene promoter methylation was continuously increased from normal endometrium, atypical hyperplasia, and EC, which was 8.3%, 18.2%, and 31.4%, respectively [28]. According to Sasaki et al., methylation of the PRB gene was detected in 74% of EC but not in normal samples [47]. Li et al., revealed that hypermethylation of *RARβ2* was 75.0% detected in endometrial hyperplasia, 92.2% in EC, and 0% in normal endometria, providing evidence that hypermethylation of the RARβ2 gene may be an early event during endometrial carcinogenesis [48]. Although related research about these genes’ promoter methylation in EC is limited, the roles of hypermethylation of these genes in endometrial carcinogenesis deserve further investigation.

## 3. Oncogenes

It is well known that oncogenes play an important role in tumorigenesis, and the activation of oncogenes can promote tumor development and even correlate with poor prognosis. A great number of studies have found that excessively expressed oncogenes play an active role in the advancement of ECs by interacting with associated signaling pathways [49,127]. Few studies, however, have demonstrated hypomethylation of oncogenes in EC. *BMP* (Bone morphogenetic protein), *CTCFL* (CCCTC-binding factor-like protein), *PARP1* (Poly (ADP-ribose) polymerase 1), *CASP8* (Caspase-8), *PAX2* (Paired box 2), *NCAPH* (non-SMC condensin I complex subunit H), and *MCM* (Minichromosome maintenance) are some examples.

Hsu et al., demonstrated that when primary ECs with recurrence were compared to tumors without recurrence, BMP genes were substantially hypomethylated in the recurrence group, and with a shorter disease-free interval [49]. Hoivik et al., showed the expression of CTCFL was discovered to rise with the development and progression of EC. The overexpression of CTCFL was significantly associated with poor survival, and loss of methylation was linked to abnormal CTCFL expression [50]. A study by Bi et al., found, in EC samples, that PARP1 mRNA and proteins were overexpressed and the PARP1 gene promoter was significantly hypomethylated; there was a strong negative connection between PARP1 mRNA levels and the number of methylated sites [51]. *CASP8* also had a similar characteristic in ECs [52]. The role of *PAX2* in ECs is controversial. According to several studies, *PAX2* was described as an oncogene involved in the development of EC [53], but others reported that it was a tumor suppressor gene [128]. What is more interesting is that the methylation status of *PAX2* is also inconsistent in EC. Two different studies reported that expression of PAX2 was increased in ECs and also demonstrated that PAX2 enhanced cell motility and boosted cell proliferation in EC cell lines. However, one of them reported that the *PAX2* promoter hypomethylation was seen in 75% of the EC samples [53], and the other one showed that the overexpression of *PAX2* was linked to the promoter hypermethylation [129]. More research is necessary to further clarify the mechanism of PAX2 overexpression in the EC. Recently, some new oncogenes were found to play a contributing role in EC progression. For instance, *NCAPH* and *MCM*. Qiu et al., first reported that the mRNA of NCAPH was overexpressed in EC and was associated with unfavorable clinicopathologic features and a bad prognosis. Upregulation of NCAPH expression was significantly associated with hypomethylation [54]. Hua and colleagues used a variety of databases to describe a series of changes in *MCM* in EC, and they concluded that the mRNA and protein levels of MCMs were greatly increased in individuals with EC, while the average methylation level in the promoter region of *MCM* was dramatically reduced [55].

In conclusion, the activation of oncogenes promotes cancer occurrence and accelerates cancer progression. Their aberrant expression usually predicts a poor prognosis. The molecular machinery involved in hypomethylation at specific oncogenes is critical for identifying the precise carcinogenic mechanisms of EC and will be valuable for EC prevention and diagnosis, risk assessment, prognosis evaluation, and the development of precise therapeutic regimens that target oncogenes. More studies on oncogene changes in EC should be conducted.

## 4. Conclusions

Epigenetics, especially DNA methylation, is now providing novel and extremely promising techniques to discover specific biomarkers and their subsequent screening. Recent studies on DNA methylation in EC provide important evidence for understanding endometrial tumorigenesis. Those findings provide potential diagnostic biomarkers and therapeutic targets. However, there are still many inconsistencies about the results of the aberrant DNA methylation within these tumor suppressor genes in EC. In the analysis of possible causes for the discordancy, the most primary reasons were sample size. Human samples feature dramatic variations in genetic, environmental, lifestyle, and individual differences. In many studies, the sample size was generally too small, owing to the huge variation in EC patients. Future research is urgently needed to address these controversies with analysis of large sample sizes. Based on the evidence in this review, the treatment of targeted DNA methylation inhibitors in EC therapy has broad application prospects. For individuals with EC, DNA methylation inhibitors have been the subject of several preclinical studies. However, no human clinical trials for this class of medications have yet been conducted [130]. Most research about the applications of DNA methylation inhibitors mainly focuses on EC cell lines. Clinical trials with demethylating agents for cervical and ovarian cancer are in progress [131,132]. It could serve as a model for clinical trials of demethylating drugs in EC patients. In further study, the nonspecific and myelotoxic properties of DNA methylation inhibitors and the heterogeneity of EC may pose challenges for relevant drug development. In addition, more research is needed to further demonstrate the relationship between aberrant methylation of target genes and the carcinogenic process and the association between the DNA methylation changes of different tumor suppressor genes, and if there are any other variables affecting methylation. Besides DNA methylation, histone modification and non-coding RNAs also play an important role in EC diagnosis and treatment. For example, Li et al., reported that the expression levels of H3K27me3 and H3K4me2 are low in the stroma of EC, and the low expression of H3K4me2 is associated with poor prognosis [133]. Oki et al., demonstrated that EC is associated with EZH2 (histone methyltransferase) overexpression and suggested that EZH2 may be a suitable therapeutic target for EC patients [134]. Cavaliere et al., summarized the role of non-coding RNAs in EC pathogenesis and prognosis in order to create personalized medicine and cancer surveillance for EC patients [135]. Further research is necessary to determine the role of DNA methylation or other epigenetic pathways in the diagnostic biomarkers and therapeutic targets of EC patients. In conclusion, studies of DNA methylation continue to provide a rich and complicated picture of epigenetic gene regulation in EC, as well as potential therapeutic targets for EC therapy.

## Figures and Tables

**Table 1:** Tumor-related genes with aberrant DNA methylation in EC.

Gene	Alternate Gene Name	Methylation Locations	References
Hypermethylated genes			
*RASSF1A*	Ras association domain family 1 isoform A	Promoter	[19,20,21,22,23,24,25]
*p16*	Cyclin-dependent kinase inhibitor 2A	Promoter	[26,27,28,29]
*hMLH1*	Human mutL homolog 1	Promoter	[20,26,30,31,32]
*PTEN*	Phosphatase and tensin homolog	Promoter	[20,33,34]
*APC*	Adenomatous polyposis coli	Promoter	[35,36,37,38,39,40]
*E-cadherin*	Epithelial cadherin	Promoter	[22,36,37,41,42]
*CDH13*	Cadherin-13	Promoter	[20,35,43,44]
*ESR1*	Estrogen receptor 1	Promoter	[45,46]
*O6-MGMT*	O6-methylguanine-DNA methyltransferase	Promoter	[28,35]
*PRs*	Progesterone receptors	Promoter	[46,47]
*RARβ2*	Retinoic acid receptor β2	Promoter	[48]
Hypomethylated genes			
*BMP*	Bone morphogenetic protein	Promoter	[49]
*CTCFL*	CCCTC-binding factor-like protein	Promoter	[50]
*PARP1*	Poly (ADP-ribose) polymerase 1	Promoter	[51]
*CASP8*	Caspase-8	Promoter	[52]
*PAX2*	Paired box 2	Promoter	[53]
*NCAPH*	non-SMC condensin I complex subunit H	Promoter	[54]
*MCM*	Minichromosome maintenance	Promoter	[55]

**Table 2:** Analysis of published data concerning methylation profiles in EC.

References	Frequencies of Gene Methylation										
	*RASSF1A*	*p16*	*hMLH1*	*PTEN*	*APC*	*E-cadherin*	*CDH13*	*ESR1*	*O6-MGMT*	*PRs*	*RARβ2*
[65]	30~50%	/	/	/	/	/	/	/	/	/	/
[24,25,66]	51~70%	/	/	/	/	/	/	/	/	/	/
[19,20,21,22,23,67]	71~90%	/	/	/	/	/	/	/	/	/	/
[20,29,35,69]	/	0~20%	/	/	/	/	/	/	/	/	/
[24,28,70,71]	/	21~40%	/	/	/	/	/	/	/	/	/
[26,27]	/	75~95%	/	/	/	/	/	/	/	/	/
[20,70]	/	/	10~30%	/	/	/	/	/	/	/	/
[72]	/	/	31~50%	/	/	/	/	/	/	/	/
[26,31,32]	/	/	61~85%	/	/	/	/	/	/	/	/
[20]	/	/	/	1~10%	/	/	/	/	/	/	/
[33,34]	/	/	/	11~30%	/	/	/	/	/	/	/
[28,34,37,38]	/	/	/	/	10~30%	/	/	/	/	/	/
[35,39,73]	/	/	/	/	31~50%	/	/	/	/	/	/
[36,40]	/	/	/	/	51~70%	/	/	/	/	/	/
[37,74,75]	/	/	/	/	/	0~20%	/	/	/	/	/
[36,42,67,76]	/	/	/	/	/	21~40%	/	/	/	/	/
[22]	/	/	/	/	/	41~60%	/	/	/	/	/
[41]	/	/	/	/	/	61~85%	/	/	/	/	/
[20,35,43,44]	/	/	/	/	/	/	61~90%	/	/	/	/
[20]	/	/	/	/	/	/	/	0~10%	/	/	/
[46]	/	/	/	/	/	/	/	40~50%	/	/	/
[45]	/	/	/	/	/	/	/	90~100%	/	/	/
[35]	/	/	/	/	/	/	/	/	0~10%	/	/
[28]	/	/	/	/	/	/	/	/	30~40%	/	/
[46]	/	/	/	/	/	/	/	/	/	20~30%	/
[47]	/	/	/	/	/	/	/	/	/	70~80%	/
[48]	/	/	/	/	/	/	/	/	/	/	90~100%

**Table 3:** The frequency of several tumor-related genes’ promoter methylation status in different histologic tissue. NE, normal endometrium; HE, hyperplasia endometrium; EC, endometrial cancer.

	Frequencies of Gene Methylation in Different Histologic Tissue			
	NE	HE	EC	Reference
*RASSF1A*	30%	36.8%	85.4%	[67]
	36%	50%	74%	[23]
*p16*	10%	7.7%	38.2%	[28]
	0%	16.7%	34.21%	[71]
*APC*	33.3%	42.4%	19.6%	[28]
	0%	7.2%	22%	[37]
*E-cadherin*	20%	21.1%	31.4%	[67]
	0%	0%	22%	[36]
	0%	0%	14%	[37]
	0%	0%	36.6%	[76]
	0%	0%	38.5%	[42]
*CDH13*	14.81%	40.58%	81.36%	[44]
*O6-MGMT*	8.3%	18.2%	31.4%	[28]
*RARβ2*	0%	75%	92.2%	[48]

## Data Availability

Not applicable.
